# BEAST: Bayesian evolutionary analysis by sampling trees

**DOI:** 10.1186/1471-2148-7-214

**Published:** 2007-11-08

**Authors:** Alexei J Drummond, Andrew Rambaut

**Affiliations:** 1 Bioinformatics Institute, University of Auckland, Auckland, New Zealand; 2 Department of Computer Science, University of Auckland, Auckland, New Zealand; 3 Institute of Evolutionary Biology, University of Edinburgh, Edinburgh, UK

## Abstract

**Background:**

The evolutionary analysis of molecular sequence variation is a statistical enterprise. This is reflected in the increased use of probabilistic models for phylogenetic inference, multiple sequence alignment, and molecular population genetics. Here we present BEAST: a fast, flexible software architecture for Bayesian analysis of molecular sequences related by an evolutionary tree. A large number of popular stochastic models of sequence evolution are provided and tree-based models suitable for both within- and between-species sequence data are implemented.

**Results:**

BEAST version 1.4.6 consists of 81000 lines of Java source code, 779 classes and 81 packages. It provides models for DNA and protein sequence evolution, highly parametric coalescent analysis, relaxed clock phylogenetics, non-contemporaneous sequence data, statistical alignment and a wide range of options for prior distributions. BEAST source code is object-oriented, modular in design and freely available at  under the GNU LGPL license.

**Conclusion:**

BEAST is a powerful and flexible evolutionary analysis package for molecular sequence variation. It also provides a resource for the further development of new models and statistical methods of evolutionary analysis.

## Background

Evolution and statistics are two common themes that pervade the modern analysis of molecular sequence variation. It is now widely accepted that most questions regarding molecular sequences are statistical in nature and should be framed in terms of parameter estimation and hypothesis testing. Similarly it is evident that to produce models that accurately describe molecular sequence variation an evolutionary perspective is required.

The BEAST software package is an ambitious attempt to provide a general framework for parameter estimation and hypothesis testing of evolutionary models from molecular sequence data. BEAST is a Bayesian statistical framework and thus provides a role for prior knowledge in combination with the information provided by the data. Bayesian Markov chain Monte Carlo (MCMC) has already been enthusiastically embraced as the state-of-the-art method for phylogenetic reconstruction, largely driven by the rapid and widespread adoption of **MrBayes **[1]. This enthusiasm can be attributed to a number of factors. Firstly, Bayesian methods allow the relatively straightforward implementation of extremely complex evolutionary models. Secondly, there is an often erroneous perception that Bayesian estimation is "faster" than heuristic optimization based on a maximum likelihood criterion.

In addition to phylogenetic inference, a number of researchers have recently developed Bayesian MCMC software for coalescent-based estimation of demographic parameters from genetic data [2-7]. Like phylogenetic analysis, these also require a gene tree in the underlying model, although in this setting, the sequences represent different individuals from the same species, rather than from different species. Most recently, Bayesian MCMC has also been applied to a central problem in evolutionary bioinformatics: the co-estimation of phylogeny and sequence alignment [8,9]. Taken together with progress in phylogenetics and coalescent-based population genetics, Bayesian MCMC has been applied to most of the evolutionary questions commonly asked of molecular data.

BEAST can be compared to a number of other software packages with similar goals, such as **MrBayes **[1], which currently focuses on phylogenetic inference and **Batwing **[4] which focuses predominantly on coalescent-based population genetics of microsatellites. Like these software packages, the core algorithm implemented in BEAST is Metropolis-Hastings MCMC [10,11]. MCMC is a stochastic algorithm that produces sample-based estimates of a target distribution of choice. For our purposes the target distribution is the posterior distribution of a set of evolutionary parameters given a set of molecular sequences. Possibly the most distinguishing feature of BEAST is its firm focus on calibrated phylogenies and genealogies, that is, rooted trees incorporating a time-scale. This is achieved by explicitly modeling the rate of molecular evolution on each branch in the tree. On the simplest level this can be a uniform rate over the entire tree (i.e., the molecular clock model [12]) with this rate known in advance or estimated from calibration information. One of the most promising recent advances in molecular phylogenetics has been the introduction of *relaxed molecular clock *models that do not assume a constant rate across lineages [13-20]. BEAST was the first software package that allows inference of the actual phylogenetic tree under such models [21].

The purpose behind the development of BEAST is to bring a large number of complementary evolutionary models (substitution models, insertion-deletion models, demographic models, tree shape priors, relaxed clock models, node calibration models) into a single coherent framework for evolutionary inference. This building-block principle of constructing a complex evolutionary model out of a number of simpler model components provides powerful new possibilities for molecular sequence analysis. The motivation for doing this is (1) to avoid the unnecessary simplifying assumptions that currently exist in many evolutionary analysis packages and (2) to provide new model combinations and a flexible system for model specification so that researchers can tailor their evolutionary analyses to their specific set of questions.

The ambition of this project requires teamwork, and we hope that by making the source code of BEAST freely available, the range of models implemented, while already large, will continue to grow and diversify.

## Results and Discussion

BEAST provides considerable flexibility in the specification of an evolutionary model. For example, consider the analysis of a multiple sequence alignment of coding DNA. In a BEAST analysis, it is possible to allow each codon position to have a different substitution rate, a different amount of rate heterogeneity among sites, and a different amount of rate heterogeneity among branches, whilst sharing the same intrinsic ratio of transitions to transversions with the other codon positions. In fact, any or all parameters (including the tree itself) can be shared or independent among partitions of the sequence data.

An unavoidable feature of Bayesian statistical analysis is the specification of a prior distribution over parameter values. This requirement is both an advantage and a burden. It is an advantage because relevant knowledge such as palaeontological calibration of phylogenies is readily incorporated into an analysis. However, when no obvious prior distribution for a parameter exists, a burden is placed on the researcher to ensure that the prior selected is not inadvertently influencing the posterior distribution of parameters of interest.

In BEAST, all parameters (whether they be substitutional, demographic or genealogical) can be given informative priors (e.g. exponential, normal, lognormal or uniform with bounds, or combinations of these). For example, the age of the root of the tree can be given an exponential prior with a pre-specified mean.

### The model of evolution

The evolutionary model for a set of aligned nucleotide or amino acid sequences in BEAST is divided into five components. For each of these a range of possibilities are offered and thus a large number of unique evolutionary models can easily be constructed. These components are:

• Substitution model – The substitution model is a homogeneous Markov process that defines the relative rates at which different substitutions occur along a branch in the tree.

• Rate model among sites – The rate model among sites defines the distribution of relative rates of evolutionary change among sites.

• Rate model among branches – The rate model among branches defines the distribution of rates among branches and is used to convert the tree, which is in units of time, to units of substitutions. These models are important for divergence time estimation procedures.

• Tree – a model of the phylogenetic or genealogical relationships of the sequences.

• Tree prior – The tree prior provides a parameterized prior distribution for the node heights (in units of time) and tree topology.

### Substitution models and rate models among sites

For nucleotide data, all of the models that are nested in the general time-reversible (GTR) model [22,23] -including the well known HKY85 model [24] – can be specified. For the analysis of amino acid sequence alignments any of the following replacement models can be used: Blosum62, CPREV, Dayhoff, JTT, MTREV and WAG. When nucleotide data represents a coding sequence (i.e. an in-frame protein-coding sequence with introns removed) the Goldman and Yang model [25] can be used to model codon evolution. In addition, Γ-distributed rates among sites [26,27] or a proportion of invariant sites, or a combination of the two [28,29] can be used to describe rate heterogeneity among sites.

### Rate models among branches, divergence time estimation and time-stamped data

The basic model for rates among branches supported by BEAST is the strict molecular clock model [12], calibrated by specifying either a substitution rate or the date of a node or set of nodes. In this context, dates of divergence for particular clades can be estimated. The clades can be defined either by an enforced grouping of taxa or as the most recent common ancestor of a set of taxa of interest. The second alternative does not require monophyly of the selected taxa with respect to the rest of the tree. Furthermore, when the differences in the dates associated with the tips of the tree comprise a significant proportion of the age of the entire tree, these dates can be incorporated into the model providing a source of information about the overall rate of evolutionary change [3,30,31].

In BEAST, divergence time estimation has also been extended to include *relaxed phylogenetics *models, in which the rate of evolution is allowed to vary among the branches of the tree. In particular we support a class of uncorrelated relaxed clock branch rate models, in which the rate at each branch is drawn from an underlying distribution such as exponential or lognormal [21].

If the sequence data are all from one time point, then the overall evolutionary rate must be specified with a strong prior. The units implied by the prior on the evolutionary rate will determine the units of the node heights in the tree (including the age of the most recent common ancestor) as well as the units of the demographic parameters such as the population size parameter and the growth rate. For example, if the evolutionary rate is set to 1.0, then the node heights (and root height) will be in units of mutations per site (i.e. the units of branch lengths produced by common software packages such as **MrBayes **3.0). Similarly, for a haploid population, the coalescent parameter will be an estimate of *N*_*e*_*μ*, where *N*_*e *_is the effective population size and *μ *is the rate of mutation per generation. However, if, for example, the evolutionary rate is expressed in mutations per site per year, then the branches in the tree will be in units of years. Furthermore the population size parameter of the demographic model will then be equal to *N*_*e*_*τ*, where *τ *is the generation length in years. Finally, if the evolutionary rate is expressed in units of mutations per site per generation then the resulting tree will be in units of generations and the population parameter of the demographic model will be in natural units (i.e. will be equal to the effective number of reproducing individuals, *N*_*e*_).

### Tree Priors

When sequence data has been collected from a homogenous population, various coalescent [32,33] models of demographic history can be used in BEAST to model population size changes through time. At present the simple parametric models available include constant size *N*(*t*) = *N*_*e *_(1 parameter), exponential growth *N*(*t*) = *N*_*e*_*e*^-*gt *^(2 parameters) and logistic growth (3 parameters).

In addition, the highly parametric Bayesian skyline plot [34] is also available, but this model can only be used when the data are strongly informative about population history. All of these demographic models are parametric priors on the ages of nodes in the tree, in which the hyperparameters (e.g., population size, *N*_*e*_, and growth rate, *g*) can be sampled and estimated. As well as performing single locus coalescent-based inference, two or more unlinked gene trees can be simultaneously analyzed under the same demographic model. Sophisticated multi-locus coalescent inference can be achieved by allocating a separate overall rate and substitution process for each locus, thereby accommodating loci with heterogeneous evolutionary processes.

At present there are only a limited number of options for non-coalescent priors on tree shape and branching rate. Currently a simple Yule prior on birth rate of new lineages (1 parameter) can be employed. However, generalized birth-death tree priors are under development.

In addition to general models of branching times such as the coalescent and Yule priors, the tree prior may also include specific distributions and/or constraints on certain node heights and topological features. These additional priors may represent other sources of knowledge such as expert interpretation of the fossil record. For example, as briefly noted above, each node in the tree can have a prior distribution representing knowledge of its date. This method of calibrating a tree based on specifying the date of one of the nodes has a long history [35]. A recent paper on "relaxed phylogenetics" contains more information on calibration priors [21].

### Insertion-deletion models

Finally, BEAST also has a model of the insertion-deletion process. This provides the ability to co-estimate the phylogeny and the multiple sequence alignment. Currently only the TKF91 model of insertion-deletion [36] is available. Interested readers should refer to the paper on this subject for more details [8].

### Multiple data partitions and linking and unlinking parameters

BEAST provides the ability to analyze multiple data partitions simultaneously. This is useful when combining multiple genes in a single multi-locus coalescent analysis (e.g. [37]) or to allocate different evolutionary processes to different regions of a sequence alignment (such as the codon positions; e.g. [6]). The parameters of the substitution model, the rate model among sites, the rate model among branches, the tree, and the tree prior can all be 'linked' or 'unlinked' in a analysis involving multiple partitions. For example in an analysis of HIV-1 group O by Lemey *et al *[37], three loci (gag, int, pol) were assumed to share the same substitution model parameters (GTR), as well as sharing the same demographic history of exponential growth. However they were assumed to have different shape parameters for Γ-distributed rate heterogeneity among sites, different rate parameters for the strict molecular clock and the three tree topologies and sets of divergence times were also assumed to be independent and unlinked.

### Model comparison and model selection

The most sound theoretical framework for model comparison in a Bayesian framework is calculation of the Bayes factor (BF):

$$BF=\frac{p(D|M_{1})}{p(D|M_{2})}$$

where *p*(*D*|*M*) is the marginal likelihood of model M, averaged over the model parameter values *θ*:

p(D|M)=∫Pr(D|θ,M)p(θ|M)dθ

So the BF is the ratio of the marginal likelihoods of the two models. Generally speaking calculating the BF involves a reversible jump MCMC in which a Markov chain is constructed that samples a state space containing both models. Reversible jump MCMC has not been implemented in BEAST yet. However there are a couple of methods of approximating the marginal likelihood of a model (and therefore the BF between two models) by processing the output of a BEAST analysis. A simple method first described by Newton and Raftery [38] computes the BF via importance sampling (with the posterior as the importance distribution). With this importance distribution it turns out that the harmonic mean of the sampled likelihoods is an estimator of the marginal likelihood:

$$m_{HM}(D|M)=(\frac{1}{N}\sum \frac{1}{Pr(D|\theta^{\left(i\right)},M)}{)}^{-1};\theta^{\left(i\right)}\sim p(\theta |D,M)$$

This estimator does not always behave very well, but there are number of modifications that can be used to stabilize it and bootstrapping can be employed to assess the uncertainty in the estimated marginal likelihoods. In general, a BF > 20 is strong support for the favoured model (*M*_1 _in equation 1).

### Example

We demonstrate some of the key features of a Bayesian analysis on a sample of 17 dengue virus serotype 4 sequences, isolated at dates ranging from 1956 to 1994 (see [30] for details). Like many RNA viruses, dengue virus evolves at a rapid rate and as a result the sampling times of the 17 isolates can be used by BEAST as calibrations to estimate the overall substitution rate and the divergence times in years. We analyzed the data under both a codon-position specific substitution model (GTR + CP), in which each codon position had a separate relative substitution rate parameter, as well as the standard GTR + Γ + I model. Both of these models have the same number of free parameters. We also investigated two different models of rates variation among branches: the strict clock and the uncorrelated lognormal-distributed relaxed molecular clock. We used the constant population size coalescent as the tree prior. For each model the MCMC was run for 10,000,000 steps and sampled every 500 steps. The first 100,000 steps of each run were discarded as burnin. This resulted in effective sample sizes for the posterior probability of much more than 1000 for all four analyses, (see Additional files 1, 2, 3 and 4, for BEAST XML input of all four runs).

**Figure 1:**
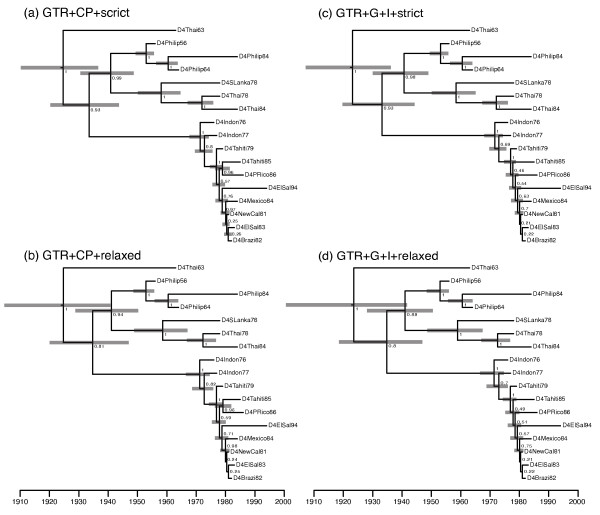
**Consensus tree of 17 dengue 4 *env *sequences **The consensus tree for the example analysis of Dengue 4 sequences under the strict clock analysis with a GTR + CP substitution model. Each internal node is labeled with the posterior probability of monophyly of the corresponding clade. The gray bars illustrated the extent of the 95% highest posterior density intervals for each divergence time. The scale is in years.

As has been previously suggested to be generally the case for protein-coding sequences [39], we found that the codon-position-specific model of rate heterogeneity among sites has a substantially superior fit to the data than the GTR + Γ + I model (see Table 1), and also supports a different consensus tree topology (see Figure 1). However we find little difference (log BF = 0.8) between the two models of rate variation among branches, indicating that this particular data can be treated as clock-like, as has been previously suggested [30]. Under the strict clock model with codon-position rate heterogeneity and a constant-size coalescent tree prior the estimated date of the root of the phylogeny is 1924 (95% highest posterior density (HPD): 1911 – 1936) and the estimated rate of substitution for this serotype was estimated to be 8.38 × 10^-4 ^(95% HPD: 6.40 × 10^-4 ^– 1.05 × 10^-3^).

**Table 1:** Summary of the four models analyzed

Substitution Model	Marginal Likelihood	50% credible set size	Mean tree height (years)
(a) GTR + CP + strict	-3656.13 ± 0.11	38	70.1 ± 0.09
(b) GTR + CP + relaxed	-3655.33 ± 0.11	57	70.5 ± 0.2
(c) GTR + Γ + I + strict	-3751.37 ± 0.11	289	71.7 ± 0.1
(d) GTR + Γ + I + relaxed	-3750.23 ± 0.11	469	72.0 ± 0.2

The marginal likelihoods, the number of distinct tree topologies in the 50% credible set and the mean tree height (± stderr) of the four substitution models that were analyzed in the example. The large improvement in marginal likelihood clearly indicates that the two codon-position substitution models (CP) are substantially superior to the models in which rate heterogeneity among sites is modeled by a 3-distribution and a proportion of invariant sites. In contrast, in this example there is little difference in fit to the data between the strict clock and the relaxed clock analyses, suggesting that this data is clock-like.

One method of summarizing the posterior distribution of phylogenetic trees is to rank the tree topologies by posterior probability and consider the smallest set of trees that represents at least *x*% of the posterior probability. This set is termed the *x*% credible set of tree topologies [40]. For the purposes of hypothesis testing, a phylogeny can be rejected if it is not found in the 95% credible set of tree topologies. In this example we found that the size of the credible sets varied substantially for the different models. In table 1 we list posterior estimates of the size of the 50% credible sets for each of the four models. We chose 50% because both the GTR + Γ + I models sampled many singleton trees in the tail of the distribution so that an accurate estimate of the size of the 95% credible set was not feasible. Nevertheless the table clearly indicates that the posterior distribution of the GTR + CP models is almost an order of magnitude more concentrated in tree space. This suggests that, for this data set, the GTR model is both a more precise estimator and a better fit to the data. In the case of the GTR + CP + strict model, 38 of the 1.1919 × 10^17 ^rooted trees with 17 tips commanded half the total probability given the data.

## Conclusion

BEAST is a flexible analysis package for evolutionary parameter estimation and hypothesis testing. The component-based nature of model specification in BEAST means that the number of different evolutionary models possible is very large and therefore diffcult to summarize. However a number of published uses of the BEAST software already serve to highlight the breadth of application the software enjoys [6,8,34,37,41].

BEAST is an actively developed package and enhancements for the next version include (1) birth-death priors for tree shape (2) faster and more flexible codon-based substitution models (3) the structured coalescent to model subdivided populations with migration (4) models of continuous character evolution and (5) new relaxed clock models based on random local molecular clocks.

## Methods

The overall architecture of the BEAST software package is a file-mediated pipeline. The core program takes, as input, an XML file describing the data to be analyzed, the models to be used and technical details of the MCMC algorithm such as the proposal distribution (operators), the chain length and the output options. The output of a BEAST analysis is a set of tab-delimited plain text files that summarize the estimated posterior distribution of parameter values and trees.

A number of additional software programs assist in generating the input and analyzing the output:

• **BEAUti **is a software package written in Java and distributed with BEAST that provides a graphical user interface for generating BEAST XML input files for a number of simple model combinations.

• **Tracer **is a software package written in Java and distributed separately from BEAST that provides a graphical tool for MCMC output analysis. It can be used for the analysis of the output of BEAST as well as the output of other common MCMC packages such as **MrBayes **[1] and **BAli-Phy **[42].

Because of the combinatorial nature of the BEAST XML input format, not all models can be specified through the graphical interface of **BEAUti**. Indeed, the sheer number of possible combinations of models mean that, inevitably, many combinations will essentially be untried and untested. It is also possible to create models that are inappropriate or meaningless for the data being analyses. **BEAUti **is therefore intended as a way of generating commonly used and well-understood analyses. For the more adventurous researcher, and with the above warnings in mind, the XML file can be directly edited. A number of online tutorials are available to guide users on how to do this.

One of the primary motivations for providing a highly structured XML input format is to facilitate reproducibility of complex evolutionary analyses. While an interactive graphical user interface provides a pleasant user experience, it can be time-consuming and error-prone for a user to record and reproduce the full sequence of choices that are made, especially with the large array of options typically available for MCMC analysis. By separating the graphical user interface (BEAUti) from the analysis (BEAST) we accommodate an XML layer that captures the exact details of the MCMC analysis being performed. We strongly encourage the routine publication of XML input files as supplementary information with publication of the results of a BEAST analysis. Because of the non-trivial nature of MCMC analyses and the need to promote reproducibility, it is our view that the publication of the exact details of any Bayesian MCMC analysis should be made a pre-requisite for publication of all MCMC analysis results.

The output from BEAST is a simple tab-delimited plain text file format with one a row for each sample. When accumulated into frequency distributions, this file provides an estimate of the marginal posterior probability distribution of each parameter (e.g. parameters such as mutation rate, tree height and population size). This can be done using any standard statistics package or using the specially written package, **Tracer **[43]. **Tracer **provides a number of graphical and statistical ways of analyzing the output of BEAST to check performance and accuracy. It also provides specialized functions for summarizing the posterior distribution of population size through time when a coalescent model is used.

The phylogenetic tree of each sample state is written to a separate file as either NEWICK or NEXUS format. This can be used to investigate the posterior probability of various phylogenetic questions such as the monophyly of a particular group of organisms or to obtain a consensus phylogeny.

Although there is always a trade-off between a program's flexibility and its computational performance, BEAST performs well on large analyses (e.g. [41]). A Bayesian MCMC algorithm needs to evaluate the likelihood of each state in the chain and thus performance is dictated by the speed at which these likelihood evaluations can be made. BEAST attempts to minimize the time taken to evaluate a state by only recalculating the likelihood for parts of the model that have changed from the previous state. Furthermore, the core computational functions have been implemented in the C programming language. This can be compiled into a highly optimized library for a given platform providing an improvement in speed. If this library is not found, BEAST will use its Java version of these functions, thereby retaining its platform-independence.

## Authors' contributions

AJD and AR designed and implemented all versions of BEAST up to the current (version 1.4.6), which was developed between June 2002 and October 2007. Portions of the BEAST source code are based on an original Markov chain Monte Carlo program developed by AJD (called MEPI) during his PhD at Auckland University between the years 2000 and 2002. Portions of the BEAST source code are based on previous C++ software developed by AR. Both authors contributed to the writing of this paper.

## Supplementary Material

Additional file 1 **Dengue4-GTR-CP-strict**. The BEAST input XML file for the GTR + CP + strict clock analysis. Click here for file

Additional file 2 **Dengue4-GTR-CP-relaxed**. The BEAST input XML file for the GTR + CP + relaxed clock analysis. Click here for file

Additional file 3 **Dengue4-GTR-GI-strict**. The BEAST input XML file for the GTR + Γ + I + strict clock analysis. Click here for file

Additional file 4 **Dengue4-GTR-GI-relaxed**. The BEAST input XML file for the GTR + Γ + I + relaxed clock analysis. Click here for file
